# Hormonal therapy after the operation for catamenial pneumothorax – is it always necessary?

**DOI:** 10.1186/s13019-016-0462-7

**Published:** 2016-04-14

**Authors:** D. Subotic, Z. Mikovic, N. Atanasijadis, M. Savic, D. Moskovljevic, D. Subotic

**Affiliations:** Clinic for thoracic surgery, Clinical Center of Serbia, Belgrade, Serbia; Clinic for gynaecology “Narodni front”, Belgrade, Serbia; University of Belgrade School of medicine, Belgrade, Serbia

## Abstract

**Background:**

Our recent clinical observations put into question the routine hormonal therapy for pneumothorax recurrence prevention, in patients operated for catamenial pneumothorax (CP).

**Methods:**

Retrospective review of the treatment of four women operated for CP in a recent 32-months period.

**Results:**

The four presented patients with CP represent 4.8 % of the overall number of patients operated for spontaneous pneumothorax and 19 % of women operated for pneumothorax in the same period. In all patients, typical multiple diaphragm holes existed. The involved part of the diaphragm was removed with diaphragm suture in three patients, whilst in one patient, a diaphragm placation was done. Endometriosis was histologically confirmed in two patients. During the follow-up period of 6–43 months, none of the patients underwent a postoperative hormonal therapy for different reasons, and in none of them the pneumothorax recurrence occurred.

**Conclusion:**

The clinical course of these patients, with the absence of the pneumothorax recurrence despite the omission of the hormonal treatment, suggests that the appropriateness of the routine hormonal treatment with gonadotrophin-releasing hormone analogues for 6–12 months, should be reconsidered and re-evaluated in further studies.

## Background

The term „catamenial pneumothorax” (CP) relates to recurrent spontaneous pneumothorax (SP) in women, whose onset has some temporal relation with menses, that is not sharply defined. According to most authors, if the onset of recurrent SP is within 72 hours after [1], or within 24 hours before and 72 hours after the onset of menses [2], the pneumothorax can be classified as catamenial.

The incidence of this predominantly right-sided disease has been underestimated for years, reported as not exceeding 3–6 % of all spontaneous pneumothoraces. Recent retrospective studies reported CP in up to one third of ovulating women operated for pneumothorax [3].

The most frequently reported mechanism of CP is a passage of air through congenital or acquired (secondary to endometriosis) diaphragmatic defects in a way that air from the vagina, through the cervix (due to absence of cervical mucous during menstruation), uterus and fallopian tubes, reaches the peritoneal cavity, and finally the pleural space through the diaphragmatic defects [4–6]. Diaphragmatic endometrial implants, if present, may cause diaphragmatic defects due to their cyclical necrosis.

There is a broad consensus that gonadotrophin-releasing hormone (GnRH) analogue therapy for 6–12 months is essential for recurrence prevention, owing to the induced hypogonadotropic hypogonadism and amenorrhea. The ovarian rest and suppression of ectopic endometrium activity achieved in that way [7, 8] prevent cyclic hormonal changes that may cause a pneumothorax.

Our clinical observation that in none of our four patients a pneumothorax recurrence occurred at follow up, despite the omission of the hormonal treatment, was the reason to reconsider the appropriatness of the routine hormonal therapy in all patients with catamenial pneumothoraces.

## Methods

In the period between October 2011. and June 2014. the diagnosis of catamenial and endometriosis-related pneumothorax was established in four women operated for recurrent spontaneous pneumothorax. In order to assess the incidence of this condition, we retrospectively reviewed hospital charts of all patients hospitally treated for spontaneous pneumothorax in the same period.

The institutional therapeutic algorythm for SP was a needle-aspiration in all patients as the initial treatment, except in patients with tension pneumothorax, spontaneous hemopneumothorax or in patients with evident major adhaesions. In case of the treatment failure, chest tube insertion and active suction for at least 3–4 days is the next step. Indications for surgery were more than two recurrences and impossibility to achieve or maintain a full lung expansion under active suction after 7-10 days at maximum. In this group of patients, we did not perform pleurectomy or chemical pleurodesis for recurrence prevention.

Surgical treatment of pneumothorax is performed by VATS. For the presented four patients, surgery was done through muscle sparing thoracotomy, because of the need for the diaphragm reconstruction.

After discharge, all operated women underwent regular gynaecological controls including ultrasonography and laboratory analyses.

The main clinical characteristics, operative findings, patohistological reports and postoperative treatment of all four patients are presented and compared with the literature data.

## Results

In the analysed period, 724 patients were admitted for SP. There were 591(81.6 %) males and 133(18.4 %) females, M:F 4.4:1. The right-sided and left-sided pneumothoraces existed in 321(54.3 %) and 263(45.7 %) patients respectively in males and in 82(61.6 %) and 50(38.4 %) patients respectively in females. Bilateral pneumothorax existed in 2 males and in one female. All five patients with spontaneous hemopneumothorax were males.

In the same period, 82/724(11.3 %) patients (61 men, 21 women) underwent a surgical treatment, either for more than two recurrences or the impossibility to achieve or maintain a full lung expansion under the active chest tube aspiration.

Among the operated patients, pneumothorax was right-sided in 38(62.3 %) and 16(76.2 %) patients and left-sided in 23(37.7 %) and 5(23.8 %) patients in men and women, respectively.

The four presented patients with CP represent 4.8 % of the overall number of operated patients and 19 % of the operated women.

The main clinical characteristics of the operated patients are summarized on Table 1.

**Table 1:** Patients’ characteristics

		Patient 1	Patient 2	Patient 3	Patient 4
Age (years)		33	33	49	37
N^o^ of SP episodes before surgery		2	5	5	2
Treatment before the OP		Needle aspiration; drainage	Needle aspirations	Drainage	Needle aspiration; drainage
Interval 1st SP episode-OP (months)		4.5	4	26	4.5
Operative finding	Diaphragm defects	Yes	Yes	Yes	Yes
	Lung bullous lesions	Yes	No	Yes	No
	Lung E.	No	No	No	No
PH confirmed endometriosis		Yes	Yes	No	No
Hormonal therapy		No	No	No	No
Follow up period (months)		24	43	14	6
Pneumothorax recurrence		No	No	No	No

### Relation with menses; number of SP recurrences

In three patients (N^o^ 2, 3 and 4), CP took place within 24–72 h of the menses onset, whilst in patient No 1, despite characteristic morphology and proven endometriosis, no temporal relation with menses existed. All patients had a chest pain at the time of pneumothorax onset, in patients N^o^ 1 and 2 associated with dyspnea and decreased effort tolerance.

The number of SP episodes in the presented patients was 2–5. In all but one patient a chest tube insertion was necessary to achieve a lung expansion. Surgery was indicated because of the impossiility to maintain lung expansion in one patient (N^o^1), whilst in two patients, the number of CP recurrences was the indication for surgery. In the 4th patient, apart from the third CP episode, surgery was indicated because of the thoracoscopically confirmed intrathoracic liver herniation through the diaphragm, radiographically manifested as undetermined ovoid opacity within the shadow of the right hemidiaphragm.

In all operated women, (including patient N^o^ 1 without temporal relation to menses), the existence of catamenial pneumothorax was anticipated before surgery (thoracoscopically confirmed in pts. N^o^ 2 and 4) and operative procedure planned accordingly.

### Operative procedure

**Fig. 1:**
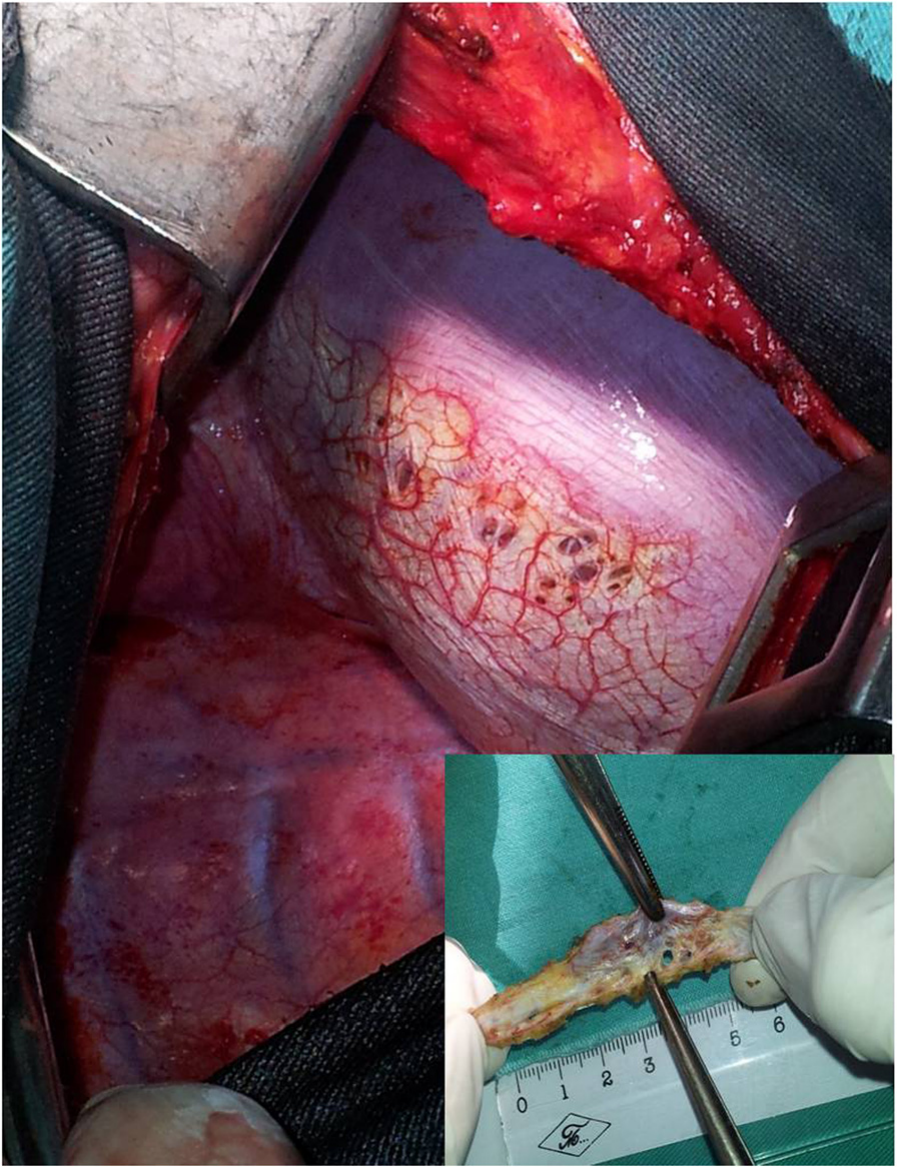
Diaphragm defects (patient 2): *insert: removed part of the diaphragm*

**Fig. 2:**
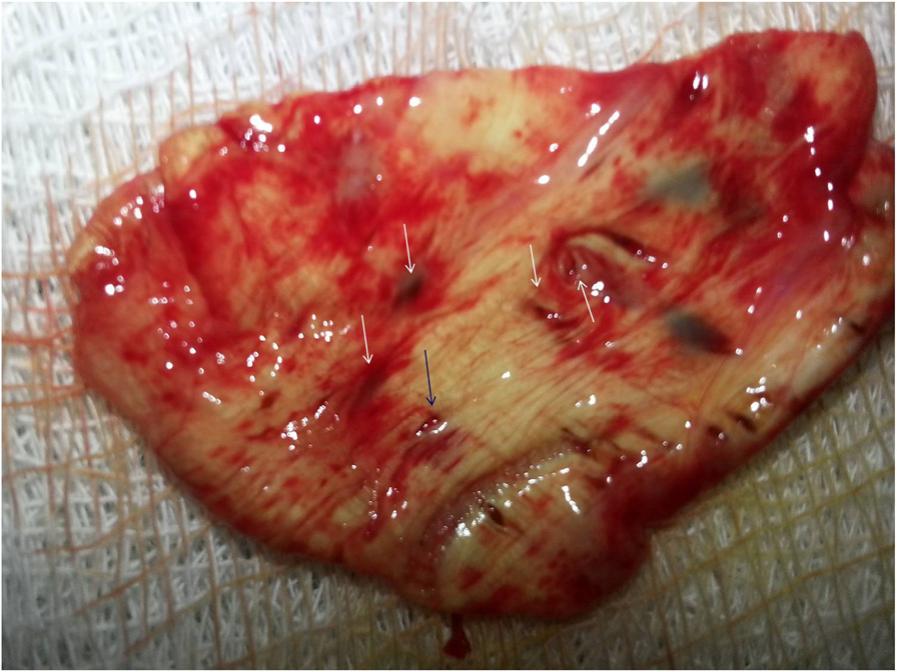
Resected part of the diaphragm (patient 1); *arrows: diaphragm defects*

**Fig. 3:**
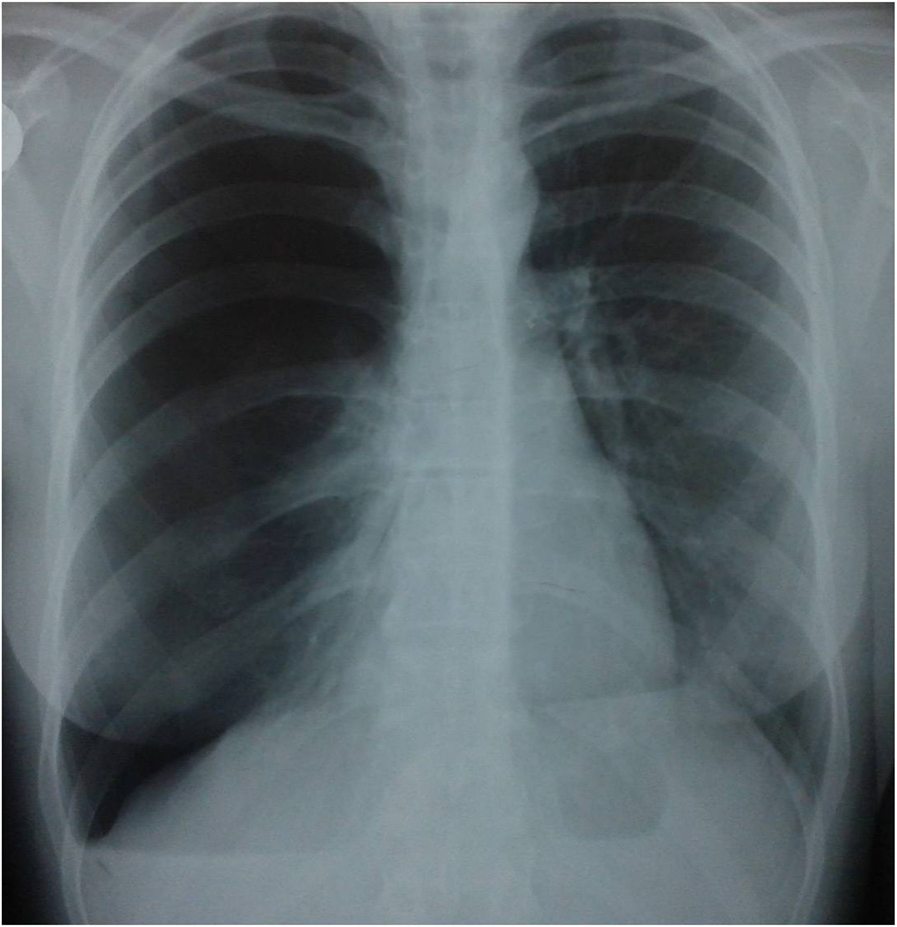
Rtg-aspect on admission (patent 1)

In all patients, operative finding was typical for CP, in form of multiple holest, 1–3 mm in size, in the membranous part of the diaphragm (Figs. 1, 2, 3). In the 1st patient, such a finding was associated with apical lung bullous lesion, whilst in the 4th patient, a large diaphragm defect with a partial liver protrusion next to the described cribriform defects existed as well.

**Fig. 4:**
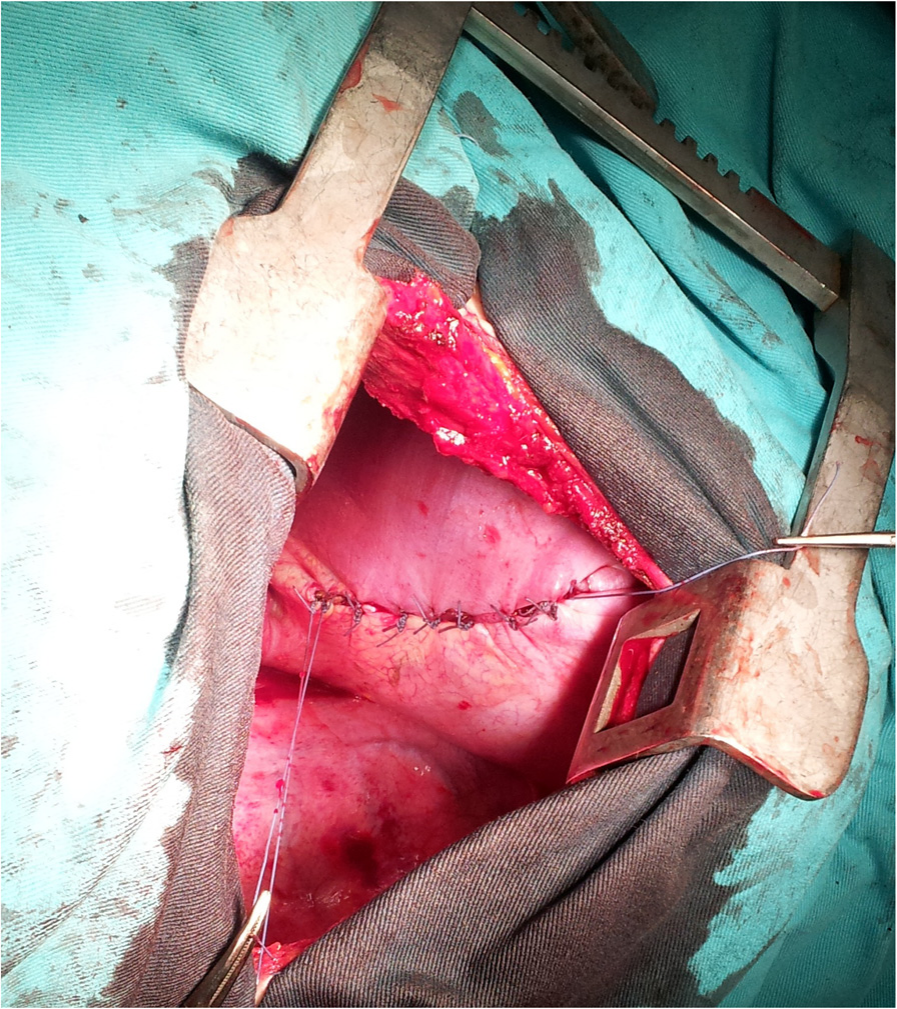
Diaphragm suture (patient 2)

In three patients, a partial excision of the diaphragm was done in a way to include all defects, followed by direct diaphragm suture (Fig. 4). In the 3rd patient, only a diaphragm plication was done. In the 1st and 3rd patients, the aformentioned procedures were associated with atypic resection of the apical lung zones, whilst in the 4th patient, a partial resection of the diaphragm preceeded the liver reposition into the abdomen.

### Follow up

None of the patients underwent a postoperative hormonal therapy for different reasons: the first patient wanted to become pregnant, whilst the second and third patients, referred to gynaecologist upon discharge, were not suggested to undergo such a treatment. Their gynaecological status and laboratory analyses were normal, with no ultrasonographic signs suggestive of endometriosis. Furthermore, coelioscopy, the only method that could reliably confirm endometriosis, was not indicated in these women. In the fourth patient, hormonal therapy was deemed unnecessary after the thorough exploration of the subdiaphragmatic region through the diaphragm defect, not revealing any signs of endometriosis.

During the follow up period of 6–43 months, in none of the four women recurrence occurred.

## Discussion

In the analysed period, the CP incidence of 0.55 % is slightly lower, but still within the reported range, not exceeding 3–6 % of all pneumothoraces [9]. On the other hand, expressed as a percentage of operated women (19 %), our case series confirms similar literature data, thus underlying the need for active diagnostic approach in women with suspicion on CP.

Before discussing possible explanations of the absence of pneumothorax relapse in the absence of postoperative hormonal therapy, some terminological clarifications should be given.

First, with the increased recognition of this disease, some confusion in the literature appeared due to the observation that in some women, pneumothorax, preceding or occurring immediately after the menses, was associated with intrathoracic endometriosis. So, from the clinical standpoint, all patients, reported under the common diagnosis of “catamenial pneumothorax”, could be divided in two groups: the first, including women in whom pneumothoraces have some temporal relation menses, and in whom proven diaphragmatic defects exist with or without endometriosis; the second group relates to so called, endometriosis-related, non-catamenial pneumothorax, ie. pneumothorax with proven intrathoracic endometriosis, with or without diaphragmatic defects, but without temporal relationship with menses [10]. Second, some authors accept the diagnosis of thoracic endometriosis only if both endometrial stroma and glands are found at histology of the lesions, and define it as “probable” when only stroma is found [11].

Our case-series, although limited, confirms the relevance of the aforementioned considerations. This because the first patient had a so called “confirmed” endometriosis, with both stroma and glands, whilst in the second patient the pathological report was highly suggestive of endometriosis, with clear endometrial stroma, but without glands. In the third patients, even in the absence of macroscopic signs of endometriosis in the vicinity of the diaphragmatic defects, it is not possible to rule it out with certainty, because the diaphragm plication was done without removing of the affected part of the diaphragm for patohistological analysis. In the fourth patient, all the SP episodes coincided with menses, whilst a big diaphragmatic defect, responsible for the absence of eventual pre-existing endometrial implants, was surrounded by small diaphragm defects, typical for CP. Those are key-arguments against the possibility of spontaneous diaphragmatic rupture in this patient, reported so far only 28 times in the English literature and being left sided in 68 % of patients [12].

The morphology of the diaphragmatic defects in patients 1–3 corresponded to those most frequently reported, in a form of holes measuring 1–3 milimeters [13], rarely more than 10 mm, [14]. Larger fenestrations (4–10 cm, or complete diaphragmatic rupture) have been only exceptionally reported [15–17]. To the best of our knowledge, the fourth presented patient is the fourth case in the English literature with CP and liver herniation through the diaphragm defect.

Although not essential for diagnosis, there are some symptoms suggestive of CP, like thoracic pain preceding or during menstruation (pts. N^0^ 2 and 3), symptoms of pelvic endometriosis, like dysmenorrhea or dyspareunia. Furthermore, history of primary or secondary infertility, with or without diagnosis of previous pelvic endometriosis, history of previous gynaecologic procedure [18], in a woman with first episode or recurrent SP should rise suspicion of this condition. The first presented patient confirms the literature data in a way that in case of SP onset in the intermenstrual period, the diagnosis of endometriosis-related pneumothorax should not be precluded, even in the absence of previous diagnosis or symptoms of pelvic endometriosis.

In all presented patients, age (generative period), pneumothorax recurrence and right-sided location were strong enough to rise suspicion of CP. Despite the fact that the correct diagnosis was obtained, in none of them hormonal therapy was given postoperatively. The reasons for such a scenario were different: the first patient wanted to be pregnant, whilst the second and third patients were not suggested hormonal treatment by their gynaecologists. In fact, the rationale for postoperative hormonal therapy - hormonal dependence of endometrial implants - with the idea to have the same effect on the pulmonary endometrial implants, could be applied to all three patients. In the first two patients the target tissue of the hormonal treatment would be the confirmed endometriosis, which, although removed by diaphragm resection, might have persisted in the vicinity or in the lungs, where they may be easily overlooked. In the third patient, even without confirmed endometriosis, hormonal treatment could have a recurrence prevention effect through preventing a passage of the air from the abdomen through tiny holes in the diaphragm in the diaphragm plication area.

It can only be speculated how long the presented patients will remain SP recurrence-free, having in mind the much higher incidence of SP recurrences in patients with catamenial vs. non-catamenial SP (32 % vs. 5.3 %) as mirrored in the experience of Alifano et al. (ref. 11). Furthermore, even postoperative recurrence rate remains quite high, as reported by Visouli (2012), Leong (2006), Marshall (2005), and Ciriaco (2009) with recurrence rates being 20 %, 25 %, 27,5 % and 40 %, respectively, during a follow up of 34–52 months [19, 20]. However, in almost all patients, recurrences occurred in patients in whom hormonal therapy was omitted or was due to be commenced. In only one series, it was not clear whether the recurrence occurred because of the absence of hormonal treatment or because of the underestimation of the tiny diaphragmatic holes that remained untreated.

Gonadotropin-releasing hormone agonists produce a hypogonadotrophic-hypogonadic state by downregulation of the pituitary gland. The treatment is restricted to 6 months, because side effects, mostly loss of trabecular bone density, hot flashes and vaginal dryness. The bone density is usually restored 2 years after treatment cessation [21, 22]. However, unlike the widely accepted period of six months of hormonal treatment as sufficient to avoid recurrences [23], there are no data about the benefit of this kind of treatment if initiated after more than 6 months or later after the operation. As presented, although none of our patients underwent a postoperative hormonal treatment, no pneumothorax recurrences were registered during a follow up of 18, 36 and 8 months. The follow up of our patients was equal or only slightly shorter than usually reported.

## Conclusion

Although not arguing against the benefit of routine postoperative hormonal therapy in all women with CP, our experience suggests that such a policy should be reconsidered and re-evaluated in further studies.
